# Improving Documentation of Side-Effects Discussions by Doctors for Psychotropic Medications in Community Settings: A Single Centre Quality Improvement Project Under the Early Intervention Service, EIS Limbrick Centre

**DOI:** 10.1192/bjo.2026.11450

**Published:** 2026-06-30

**Authors:** Mirian Okechukwu

**Affiliations:** Sheffield Health Partnership University NHS Foundation Trust, Sheffield, United Kingdom

## Abstract

**Aims:**

This quality improvement project was aimed at enhancing the clinical discussion and documentation of side effects of psychotropic medications in psychiatry community settings to 100% over a span of 12 months.

**Methods:**

This quality improvement project was carried out at the early intervention service Limbrick Centre. Sample included looking at 20 patients' first medical review under the EIS. The selection process was randomly done. Patients with a primary diagnosis of first-episode psychosis were selected using their hospital number. The audit included all patients who were referred to the EIS from 1 July to 31 December 2024. Anonymised patient data were used in line with the quality improvement initiative of this project and therefore informed consent was not applicable. Data was retrospectively obtained from outpatient electronic records over six months on patients who were newly prescribed psychotropic medications. The key standard for the baseline measurement was whether the communication of at least one side effect of a newly prescribed medication was documented by the doctor in the patient's electronic records. If electronic entries stated that side effects were 'discussed', these entries were not considered as objective evidence of discussions. The criterion for appropriate documentation was clear descriptions of specific side effects discussed and the minimum number of side effects that a doctor should discuss was set at one.

A random sampling method for data extraction was used, using the random number function in Microsoft Excel. Psychotropic medications included antidepressants, antipsychotics, benzodiazepines, mood stabilizers and hypnotics. Data was analysed using Microsoft Excel.

**Results:**

Forty percent of service users’ medical reviews had documentation of side effects of psychotropic medications. Sixty percent of service users’ medical reviews did not have documentation of side effects of psychotropic medications. Although out of this 60%, some doctors had written side effects discussed but the actual side effect discussed was not documented which therefore excluded these data.

**Conclusion:**

This project served as a reminder that robust documentation of patient communication is at the very core of good clinical practice. Shared decision making involving patient in treatment decisions and embedding robust documentation into practice, in addition to enhancing care, confers added benefits to both individual healthcare providers and their employers through the potential diminution of medico-legal risks.

